# Exclusion and Truancy of Autistic Adolescents in a UK Population Representative Sample

**DOI:** 10.1111/cch.70187

**Published:** 2025-11-21

**Authors:** Vasiliki Totsika, Kylie M. Gray, Francesca Solmi

**Affiliations:** ^1^ Division of Psychiatry University College London London UK; ^2^ Millennium Institute for Care Research, MICARE Santiago Chile; ^3^ Intellectual Disabilities Research Institute University of Birmingham Birmingham UK; ^4^ Department of Psychiatry Monash University Melbourne Australia

**Keywords:** adolescents, autism, exclusion, school non‐attendance, truancy

## Abstract

**Background:**

Autistic students experience many problems with school attendance. School exclusion and truancy are among the least researched school attendance problems in this population. The study aimed to describe levels of exclusion and truancy in a UK population‐representative sample of autistic adolescents and identify child, family and school factors associated with each school attendance problem.

**Methods:**

Data were drawn from the Millennium Cohort Study where exclusion and truancy information was available for 460 autistic 14‐year‐olds. Descriptive statistics were used to report the weighted prevalence of exclusion and truancy. A bio‐ecological framework guided the selection of available child, family and school factors potentially associated with exclusion and truancy for modelling. Univariable and multivariable logistic regression models were fitted to investigate associations.

**Results:**

Twenty‐five percent of autistic adolescents were temporarily or permanently excluded at least once. Fifteen percent of autistic adolescents reported truanting at least once. Externalising problems were strongly associated with higher odds of exclusion and truancy. The level of school support was strongly associated with higher odds of exclusion. There was weak evidence of an association between exclusion and low parental school engagement and between truancy and the absence of intellectual impairment.

**Conclusion:**

Poor mental health and in particular externalising difficulties seem to be strongly associated with a greater likelihood of parent‐reported exclusion and adolescent‐reported truancy. The role of school support and adaptation to the child's needs warrants further investigation.

## Summary

- High levels of exclusion (25%) and truancy (15%) among autistic 14‐year‐olds in this population‐representative sample highlight the urgent need for support to a vulnerable group of students.
- Strong evidence of associations with externalising problems (though not internalising) calls for prioritising support and intervention for these students.
- Future research is needed to investigate how effective school support is in preventing future school exclusion.

## 1 Introduction

Autistic children face many barriers in education (Mandy et al. 2016; Sasso and Sansour 2024). School absence levels are high; for example, in 2016–2017, 17% of autistic students in English schools missed more than 10% of school days compared to just 11% of all students (DfE 2018). School attendance problems (SAPs) encompass school absence due to school refusal, withdrawal, exclusion, truancy and absence due to ill health (Heyne et al. 2019). Of these SAPs, school exclusion and truancy in autistic students—two SAPs with significant implications for the child and the family—have received little research attention to date. The present study aims to investigate school exclusion and truancy in autistic adolescents.

In the UK educational system, school exclusion is a disciplinary measure and can be issued either for a short fixed‐term period, often called suspension, or indefinitely, often called permanent exclusion from school. As a term, it encompasses absenteeism that is initiated by the school (Kearney et al. 2019). There is limited evidence on how common school exclusion is among autistic children. In a total population study of all 7–16‐year‐old students in Wales, one of the UK's four countries, between 2 and 6% of autistic students were excluded (John et al. 2021), with the highest proportions recorded at age 14 years (6%). While this study had a robust design drawing from a whole country electronic register and school records, not all exclusions are formally recorded by schools and many autistic children are asked by schools to stay at home, a form of informal exclusion (Ambitious about Autism 2017; Power and Taylor 2020). Therefore, a prevalence of 2–6% likely underestimates the prevalence of exclusion as recent studies evidenced that autistic students are experiencing both formal and informal exclusion (Totsika et al. 2020; Totsika et al. 2023). Therefore, parent report of any formal or informal exclusion provides a more comprehensive measure of exclusion.

Two studies that used parental report of exclusion investigated factors most likely associated with school exclusion among autistic students (Totsika et al. 2020, 2023). In Totsika et al. (2020) family socioeconomic factors were the only factors significantly associated with exclusion whereas in Totsika et al. 2023, these were not. However, both studies relied on convenience sampling and these findings may be affected by selection bias. More robust sampling approaches are needed to investigate factors potentially associated with school exclusion. Qualitative evidence has suggested that exclusion often arises following difficulties the autistic child experiences in the school environment due to the lack of adaptation of the school to the child's needs as well as tensions in school relationships, including bullying (Brede et al. 2017; Gray et al. 2023; Sproston et al. 2017; Truman et al. 2021).

Truancy refers to school absence initiated by the student (Kearney et al. 2019). Truancy has been defined as school absence without permission from school and in the absence of the parents' knowledge (Heyne et al. 2019). Truancy is more pronounced in secondary school age and shows a marked increase at age 14 (Attwood and Croll 2006). Between 6.1 and 22.8% of 14–15‐year‐olds self‐reported skipping school without permission in two UK‐representative samples (Attwood and Croll 2006, 2015). To our knowledge only one study investigated truancy levels in autism and found that truancy accounted for less than 1% of days (Totsika et al. 2020). However, the study measured truancy using parent report. Given the well‐accepted definition of truancy as school absence without the parent's knowledge (Heyne et al. 2019), findings from that study contribute little to our understanding of truancy in autistic students.

Truancy has been associated with low family socioeconomic status, negative attitudes towards school, lower levels of parental monitoring, and greater exposure to bullying (Attwood and Croll 2006, 2015). Due to the lack of research into truancy in autistic students little is known about child‐, family‐ and school‐level factors potentially associated with this SAP, and in particular, any associations with mental health problems which are highly prevalent in autism (Hossain et al. 2020) and which have been associated with truancy in non‐autistic younger children (specifically, externalising problems; Barthelemy et al. 2022).

Truancy has demonstrated little continuity over time, i.e., presenting with truancy one year does not increase the probability of truanting in a different year (Attwood and Croll 2006). Such findings highlight the need to examine concurrent contextual factors to help us gain an understanding of this systemic phenomenon (Melvin et al. 2019; Sasso and Sansour 2024).

To increase our understanding of truancy and exclusion in autistic adolescents, the present study drew on data from a UK‐representative sample of 14‐year‐olds who are part of a UK birth cohort study (Millennium Cohort Study [MCS]). Our first aim was to describe levels of adolescent‐reported truancy and parent‐reported exclusion in this population‐representative sample of autistic adolescents. Our second aim was to investigate factors associated with exclusion and truancy to increase understanding of individual‐level and contextual factors associated with these SAPs. We adopted a descriptive approach, identifying candidate factors from existing literature and the KiTes model of school absence (Melvin et al. 2019). This is a bioecological model that views SAPs as a systemic phenomenon driven by factors associated with the child, family and school (in the micro and mesosystems) as well as factors relevant to time (e.g., school year) and the broader community and policy system (Melvin et al. 2019). In our study, we selected candidate factors for their likely association with exclusion and truancy and we were interested in investigating which ones would remain associated with each type of SAP when modelling all factors together. School attendance is an essential component of school participation (Imms et al. 2017), and research into factors that are related to attendance has the potential to inform our understanding of contextual factors that may promote or threaten the participation of autistic children in school and education.

## 2 Method

The MCS is a UK birth cohort study following over 19 000 children born in 2000–2002 (Connelly and Platt 2014). The present study draws on data from wave 6 when participants were 14 years old. For the identification of autistic MCS participants, data were drawn from waves 3–6, when children were 5, 7, 11 and 14 years old.

### 2.1 Participants

Participants included 460 14‐year‐old autistic adolescents who at wave 6 had data on either school exclusion or truancy (see Table 1). Most participants were male (78%) and of white ethnicity (89%). Intellectual impairment was present in 35% of participants. Over a third of participants lived in families experiencing income poverty and in single‐parent households. Most participants (76%) lived in households where at least one parent was employed.

**TABLE 1:** Sociodemographic characteristics for 460 children with ASC at age 14 years and their families.

Child characteristics	*N* = 460
Child sex male, *n* (%)	359 (78%)
Child ethnicity white, *n* (%)	405 (89%)
Child ethnicity non‐white, *n* (%)	51 (11%)
Intellectual impairment, *n* (%)	154 (35%)
SDQ externalising^a^ score, Mean (SD)	8.32 (4.88)
SDQ internalising^b^ score, Mean (SD)	8.15 (4.71)
**Household characteristics**	
Unemployed household, *n* (%)	112 (24%)
Parental education below university level, *n* (%)	234 (57%)
Income poverty, *n* (%)	178 (39%)
Single parent family, *n* (%)	162 (35%)

^a^ Strengths and Difficulties Questionnaire (SDQ) externalising score is a sum of conduct problem and hyperactivity scores.

^b^ SDQ internalising score is a sum of emotional problems and peer problem scale.

### 2.2 Measures

#### 2.2.1 Exclusion and Truancy

Exclusion was identified by parents' responses to two questions: ‘Has your child ever been temporarily excluded from school for at least one day?’ and ‘Has your child ever been expelled or permanently excluded from school?’. Parents could respond ‘yes’ or ‘no’ to each question. Exclusion was defined as any instance of ‘yes’ in either of these questions.

Truancy was measured by adolescents' responses to the question: ‘In the last 12 months, have you ever missed school without your parents’ permission even if only for half a day or a single lesson?’. Responses were ‘yes' and ‘no’, while participants were provided with an option to not respond if they did not want to. Those who did not want to respond were coded as missing.

#### 2.2.2 Measures of Child‐Related Factors

Child mental health was measured using the Strengths and Difficulties Questionnaire (SDQ; Goodman 1997). The SDQ includes 25 parent‐reported items, each scored by 0 (not true) 1 (somewhat true) 2 (certainly true). We used the SDQ's externalising (conduct problems and hyperactivity) and internalising sub‐scales (emotional problems and peer problems). Internal consistency (Cronbach's alpha) in the current sample was 0.75 for the externalising score and 0.76 for the internalising scores.

Adolescents reported on bullying victimisation and perpetration through these questions: ‘How often do other children hurt you or pick on you on purpose?’ and ‘How often do you hurt or pick on other children on purpose?’. Responses ranged from 1 (most days) to 6 (never). Risk taking was also reported directly by adolescents: ‘On a scale of 0‐10, where 0 is never and 10 is always, how willing to take risks would you say you are?’

Intellectual impairment was measured through standardised cognitive assessments conducted at ages 3, 5 and 7 years (Totsika et al. 2021). Intellectual impairment was defined as a score of at least one standard deviation below the mean (Greenspan 2017), representing a continuum of intellectual functioning that would include borderline intellectual functioning and intellectual disability, conditions that often co‐occur with autism, and increase vulnerability for adverse outcomes (Barnevik Olsson et al. 2017; Peltopuro et al. 2014).

Ethnicity information was available across six main categories (white, mixed, Indian, Pakistani and Bangladeshi, Black or Black British, other). Study participants from an ethnic minority background were few, so this variable was recoded to identify participants of white ethnicity and those from an ethnic minority background.

#### 2.2.3 Measures of the Family Environment

Maternal mental health was measured using the Kessler 6 (K6), a measure of psychological distress in the past 30 days (Kessler et al. 2002). Parental engagement with school was measured through the question: ‘Have you attended your child's parents evening?’ with a binary answer ‘yes’ or ‘no’. If the parents' evening had not taken place yet, answers were recoded as missing. We included four family socioeconomic indicators: income poverty defined as family income less than 60% of the median equivalised household income (Förster and Pearson 2002); single parent family; parental unemployment (at least one parent in the household is in paid employment vs. no parent is employed); and parental education (at least one parent in the household is educated at university degree level vs. education below university degree level).

#### 2.2.4 Measures Related to School Characteristics

The MCS included information to describe the level of support and adaptation at school. Parents were asked ‘Does your child get any support at school due to Special Educational Needs?’ Parents could select one or more of six options provided: individual support in class, special classes, adaptation to physical environment, equipment provided, or the child attends a special school. We created a count of these items, with scores ranging 0–6 with higher scores indicating a higher level of support and adaptation.

### 2.3 Procedure and Approach to Analysis

**FIGURE 1:**
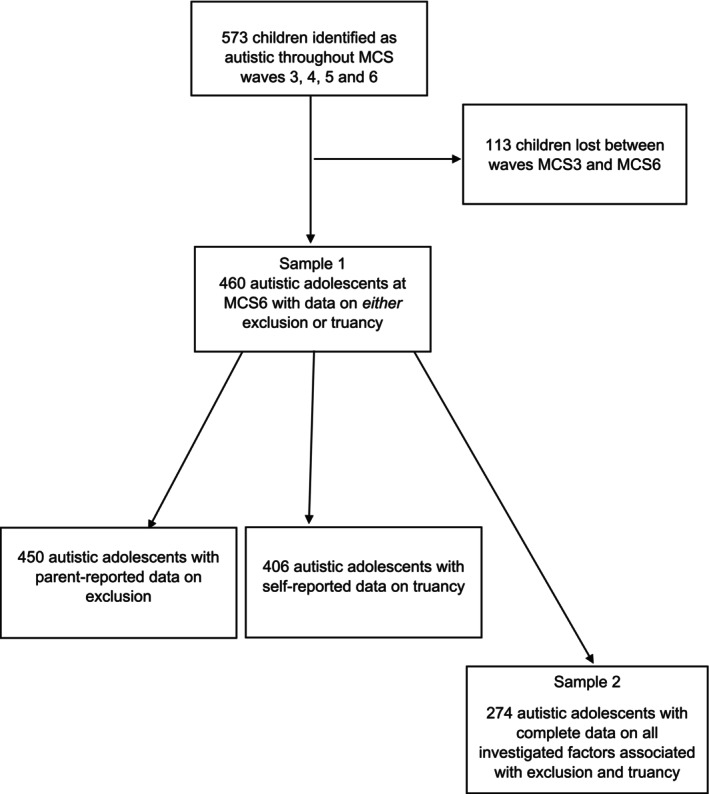
Participant selection flowchart.

We initially identified study participants by drawing on parent‐reported information across waves 3–6 about the availability of an autism diagnosis. Parents were asked ‘Has a doctor or other health professional ever told you that your child had autism, Asperger's or other autistic spectrum disorder?’. Children who were identified as autistic across any of these waves (i.e., waves 3, 4, 5 or 6) and were the primary MCS child (i.e., not a twin or a triplet) were considered for inclusion in the present study (*N* = 573). Of those, 460 also had data on exclusion or truancy at age 14 (MCS wave 6) and were included in the present study (see Figure 1 for a flowchart of participant selection). To address the first research aim, we present descriptive statistics on parent‐reported school exclusion and adolescent‐reported truancy at age 14, including weighting to account for MCS's sampling design and attrition (Fitzsimons 2017). To investigate factors potentially associated with exclusion and truancy in autism (second research aim), the analysis focused on participants who had complete data on all variables (*N* = 274). Missing data ranged from 0% (gender, SDQ scores, family poverty, single‐parent family) to 22% (bullying others). We hypothesised that data were missing at random (MAR) because in population‐based studies, unmeasured factors, including genetic factors, cannot be ruled out as responsible for attrition (Taylor et al. 2018). To account for missing data, MCS weights were used in all analyses (Fitzsimons 2017).

Each potential correlate was associated with the outcome in univariable logistic regression models to identify variables eligible for inclusion in multivariable models as the sample size was relatively small. The inclusion criterion was set to *p* ≤ 0.15 to reduce the likelihood of type II errors. Participant sex and ethnicity were retained a priori in the multivariable analyses as they represent key child characteristics. Tables 3 and 4 report adjusted odds ratios (AORs) with 95% confidence intervals (CIs) for final models.

## 3 Results

### 3.1 Levels of Parent‐Reported Exclusion and Adolescent‐Reported Truancy in Autistic Adolescents

Exclusion, as reported by parents, was present for 86 of 450 participants which represents 19% of the unweighted sample and 25% of the weighted sample. Truancy was reported by 61 of 406 responding adolescent participants representing 15% of the unweighted and 15% of the weighted sample, respectively.

### 3.2 Factors Associated With Exclusion and Truancy

Table 2 presents the ORs from univariate associations. Exclusion was strongly associated with higher SDQ externalising scores (OR 1.14, 95% CI 1.07, 1.22, *p* < 0.001), higher level of school support (OR 1.90, 95% CI 1.38, 2.64, *p* = 0.082) and income poverty (OR 2.74, 95% CI 1.43, 5.22, *p* = 0.002). Exclusion was further associated with higher SDQ internalising symptoms, higher risk taking, worse maternal mental health, lower parental engagement, parental unemployment and living in a single parent family. In the multivariable model (Table 3), there was strong evidence of an association between exclusion and higher SDQ externalising scores (AOR 1.12, 95% CI 1.03, 1.23, *p* = 0.010) as well as higher level of school support (AOR 1.73, 95% CI 1.20, 2.50, *p* = 0.004). There was weak evidence of an association between exclusion and lower levels of parental school engagement (AOR 0.36, 95% CI 0.11, 1.12, *p* = 0.077).

**TABLE 2:** Univariable analysis of potential correlates in exclusion and truancy in complete cases, *n* = 274.

	Exclusion		Truancy	
Potential correlates	OR (95% CI)	*p*	OR (95% CI)	*p*
Child is female	0.65 (0.28, 1.47)	0.297	1.47 (0.72, 3.02)	0.292
Child is from a white ethnic group	0.63 (0.25, 1.56)	0.313	1.82 (0.53, 6.28)	0.345
Child has an intellectual impairment	0.94 (0.45, 1.98)	0.876	**0.52 (0.22, 1.24)**	0.**141**
SDQ externalising	**1.14 (1.07, 1.22)**	**< 0.001**	**1.16 (1.08, 1.24)**	**< 0.001**
SDQ internalising	**1.05 (0.99, 1.13)**	0.**121**	**1.07 (1.00, 1.14)**	0.**052**
Been bullied	1.07 (0.91, 1.27)	0.412	1.10 (0.93, 1.31)	0.253
Bullying others	1.18 (0.92, 1.52)	0.187	**1.35 (1.07, 1.72)**	0.**013**
Risk taking	**1.10 (0.97, 1.25)**	0.**131**	**1.12 (0.98, 1.28)**	0.**094**
Maternal mental health (K6)	**1.06 (1.00, 1.13)**	0.**068**	**1.06 (0.99, 1.13)**	0.**089**
Parent school engagement	**0.40 (0.14, 1.12)**	0.**082**	3.65 (0.47, 28.08)	0.213
Level of school support and adaptation	**1.91 (1.38, 2.64)**	**< 0.001**	**1.42 (1.03, 1.97)**	0.**033**
Unemployed household	**2.26 (1.10, 4.64)**	0.**027**	0.97 (0.42, 2.23)	0.936
Income poverty	**2.74 (1.43, 5.22)**	0.**002**	1.23 (0.63, 2.41)	0.549
Parental education below university level	1.35 (0.71, 2.58)	0.361	**1.96 (0.99, 3.90)**	0.**053**
Single parent family	**2.20 (1.16, 4.20)**	0.**016**	1.09 (0.55, 2.16)	0.804

*Note:* Results with *p* ≤ 0.15 in bold. The alpha level was adjusted to reduce the likelihood of type II errors in this part of the analysis.

**TABLE 3:** Adjusted odds ratios (AORs) for factors associated with exclusion in the final multivariable model, *n* = 274.

Potential correlates	AOR (95% CI)	*p*
SDQ externalising	**1.12 (1.03, 1.23)**	0.**010**
SDQ internalising	0.96 (0.88, 1.06)	0.447
Risk taking (ref never)	1.07 (0.92, 1.23)	0.380
Maternal mental health (K6)	1.03 (0.95, 1.11)	0.537
Parent school engagement	0.36 (0.11, 1.12)	0.077
Level of school support and adaptation	**1.73 (1.20, 2.50)**	0.**004**
Unemployed household	0.94 (0.34, 2.56)	0.899
Income poverty	1.79 (0.78, 4.10)	0.167
Single parent family	1.49 (0.66, 3.33)	0.335

*Note:* Results with *p* < 0.05 in bold.

In the univariable models, truancy was associated with higher SDQ externalising scores (OR 1.16, 95% CI 1.08, 1.24, *p* < 0.001) and higher bullying (perpetrator) (OR 1.35, 95% CI 1.07, 1.72, *p* = 0.013). Higher odds of truancy were further associated with higher SDQ internalising scores, higher risk taking, worse maternal mental health, higher level of school support and lower parental education. The odds of truancy were lower for autistic adolescents with intellectual impairment (Table 2). In the multivariable model (Table 4), there was strong evidence for the association of truancy with higher SDQ externalising scores (AOR 1.15, 95% CI 1.05, 1.25, *p* = 0.003) and weak evidence for an association with the presence of intellectual impairment (AOR 0.41, 95% CI 0.16, 1.02, *p* = 0.055).

**TABLE 4:** Adjusted odds ratios (AOR) for factors associated with truancy in final multivariable model, *n* = 274.

Potential correlates	AOR (95% CI)	*p*
Child has an intellectual impairment	0.41 (0.16, 1.02)	0.055
SDQ externalising	**1.15 (1.05, 1.25)**	0.**003**
SDQ internalising	0.99 (0.90, 1.08)	0.784
Bullying others	1.16 (0.88, 1.53)	0.299
Risk taking	1.08 (0.93, 1.26)	0.315
Maternal mental health (K6)	1.04 (0.97, 1.13)	0.269
Level of school support and adaptation	1.30 (0.89, 1.90)	0.174
Parental education below university level	1.41 (0.67, 2.97)	0.373

*Note:* Results with *p* < 0.05 in bold.

## 4 Discussion

In this UK population representative sample, 25% of autistic 14‐year‐olds had been excluded from school, while 15% of autistic adolescents self‐reported truanting. Exclusion levels reported here are much higher than the 6% reported in a total population study in Wales of 14‐year‐old autistic students (John et al. 2021) and the 9.6% reported in a total population study of autistic students in England in 2016/17 (Hatton 2018). Unlike the John et al. (2021) and Hatton (2018) studies that measured formal exclusion from school records, exclusion in the present study included informal/unofficial exclusions, providing a more comprehensive exclusion prevalence. No comparable truancy data are available for autistic adolescents but previous UK population‐representative data estimate truancy to lie between 6.1 and 22.8% of all 14–15‐year‐olds (Attwood and Croll 2006, 2015).

When we modelled exclusion and truancy in multivariable models to simulate more closely the complex system within which they occur, there was strong evidence of adjusted associations between higher levels of externalising problems and both exclusion and truancy. Evidence from general population studies has already demonstrated a link between child mental health and school exclusion. Evidence from a UK‐representative study that examined the association between exclusion and child mental health (as total SDQ scores) indicated that for every one‐point increase in SDQ total scores, the odds of exclusion among the general population of 11–15‐year‐old students increased by 15% (Ford et al. 2018). This is similar to our study, where for every one‐point increase in SDQ externalising scores, the (adjusted) odds of exclusion increased by 12% (Table 3). Findings also align with evidence from a total Scottish population study where the co‐occurrence of ADHD with autism increased the likelihood of exclusion by 80% (Fleming et al. 2020). Our findings showed that for every one‐point increase in SDQ externalising scores, the (adjusted) odds of truancy increased by 15% (Table 4), confirming earlier evidence of an association between truancy and child mental health in a UK general population study (Attwood and Croll 2015).

In addition, findings from the adjusted models indicated an association of exclusion with a higher level of school support (strong evidence) and lower levels of parental school engagement (weak evidence). Qualitative evidence has identified a lack of school support and adaptation as a reason for the exclusion of autistic students (Brede et al. 2017; Gray et al. 2023; Sproston et al. 2017; Truman et al. 2021). In our study, findings indicated that a higher level of school support was associated with higher (adjusted) odds of exclusion. The direction of the association found in the present study was thus unexpected, but it is likely related to the fact that data are cross‐sectional and timing is concurrent: There is some evidence that school support is put in place after exclusion takes place (Gray 2018) or school attendance difficulties emerge (Nordin et al. 2023). Another possibility is that the measure of the extent of school support and adaptation around the child's special educational needs was an insufficient indicator of the quality of school support or the match between need and provision; qualitative evidence highlights that successful school support is not just about the types of supports offered but the way they are being implemented by teachers and school staff and how they are received by autistic students (Gray et al. 2023). The fact that there were higher levels of support in our study does not necessarily mean that support was of good quality or a good match to the child's needs. Future research needs to examine school support and adaptation in terms of its capacity to meet student need while longitudinal research is needed to investigate further any timing effects between school support and exclusion. The association between exclusion and parental engagement with school should be interpreted with caution both because the evidence of association was weak and because this was the first time parent‐school involvement was investigated in relation to school exclusion or school absenteeism among autistic students (c.f., Nordin et al. 2023; Sasso and Sansour 2024). A meta‐analysis of 44 risk factors for school absenteeism among non‐autistic students, identified parental involvement with school as one of 12 significant risk factors (Gubbels et al. 2019), suggesting that this is a contextual factor that warrants further research in autism. It is currently unclear how well the measure used in the current study (attendance at parents' evening at school) acted as a proxy indicator for parents' involvement with the school vs. the child's education (c.f., McNeal 2014), or indeed the quality of parent involvement. However, there is prior evidence in autism research that a positive parent–school relationship is associated with lower odds of school exclusion (Totsika et al. 2023).

Findings need to be considered in light of the study's limitations. While drawing on parent‐reported formal and informal exclusion provides a more comprehensive measure of exclusion than using only school‐reported formal exclusion, the approach still misses out on other informal exclusionary practices; for example, when students are removed from classrooms but not the school building or removed from school through managed moves between schools (Cleary et al. 2024; Power and Taylor 2020). The use of data from MCS ensured access to a population‐representative sample of autistic children. However, we cannot exclude the possibility of selection bias, if autistic children included in the present study at age 14 differed from those MCS autistic participants who had left, in relation to characteristics that were associated with the variables used in the analyses. To account for this, we have used MCS‐issued population weights also accounting for characteristics associated with losses to follow‐up in the cohort.

Overall, the present study found high levels of parent‐reported exclusion and adolescent‐reported truancy among 14‐year‐old autistic students in the UK. There was strong evidence of an association with externalising problems for both types of SAPs, highlighting a need to prioritise support for child mental health. Evidence of an association between the extent of school support for child special educational needs and higher odds of exclusion might suggest that school support is put in place around the time of exclusion or that support might be a poor match to the child's needs. Findings provide emerging evidence of child, family and school‐level factors associated with school exclusion and truancy in autistic adolescents and further replication is warranted. School attendance is a necessary first step towards school participation. Exclusion and truancy, while two very different types of SAPs, both result in the exclusion of a vulnerable group of students from school participation. Emerging findings on the role of child externalising behaviours, school support and parental involvement highlight areas future research on the promotion of school participation should explore further.

## Author Contributions

VT and FS were responsible for conceptualising the study, including designing the method and supervising the analysis. KG contributed to the design of the study and the method. VT wrote the original draft and led on reviewing and editing the manuscript. FS and KG contributed to writing, reviewing and editing the manuscript.

## Funding

The authors have nothing to report.

## Ethics Statement

All waves of the Millennium Cohort Study received ethical approval from the UK's National Health Service (NHS) Research Ethics Committees (MREC/01/6/19; MREC/03/2/022; 05/MRE02/46; 07/MRE03/32;11/YH0203;13/LO/1786). This study involved the secondary analysis of publicly available data, so further ethical approval was not needed.

## Conflicts of Interest

The authors declare no conflicts of interest.

## Data Availability

The data that support the findings of this study are openly available in UK Data Service at https://ukdataservice.ac.uk/.
